# Network Pharmacology and Molecular Docking Analysis on Molecular Targets and Mechanisms of Aidi Injection Treating of Nonsmall Cell Lung Cancer

**DOI:** 10.1155/2022/8350218

**Published:** 2022-12-06

**Authors:** Weizhou Zhang, Wenpan Peng, Yehui Li, Tingyu Pan, Fanchao Feng, Jie Xu, Xianmei Zhou

**Affiliations:** ^1^Affiliated Hospital of Nanjing University of Chinese Medicine, Nanjing 210029, China; ^2^Department of Pulmonary and Critical Care Medicine, Jiangsu Province Hospital of Chinese Medicine, Nanjing 210029, China; ^3^Department of Respiratory Medicine, Suzhou Affiliated Hospital of Nanjing University of Chinese Medicine, Suzhou 215009, China

## Abstract

**Background:**

Aidi injection (ADI) is a compound preparation injection of Chinese herbs used to treat patients of nonsmall cell lung cancer (NSCLC) in China. This study aimed to reveal the mechanism of ADI in the treatment of NSCLC by using network pharmacology and molecular docking.

**Methods:**

The related targets of ADI and NSCLC were obtained from multiple databases. The network diagram of disease-drug-components-targets (DDCT) and protein-protein interaction (PPI) was constructed to screen key targets. Then, the key targets and main signaling pathways were screened by gene ontology (GO) and Kyoto Encyclopedia of Genes and Genomes (KEGG) enrichment analysis. Next, in order to validate the results of network pharmacology, expression analysis and survival analysis of key genes were performed. Finally, we carried out the technology of molecular docking to further validate the accuracy of the above results.

**Results:**

A total of 207 targets of ADI and 5282 targets of NSCLC were obtained finally. Through the construction of DDCT and PPI network diagrams, 28 key targets were finally obtained. The results of the KEGG enrichment analysis indicated that multiple signaling pathways were associated with NSCLC, which included the MAPK signaling pathway, the IL-17 signaling pathway, and the PI3K/AKT signaling pathway. The key genes in the signaling pathway mainly include TP53, CASP3, MMP9, AKT1, PTGS2, and MAPK1. The results of differently expressed analysis of key genes showed that TP53, CASP3, MMP9, AKT1, PTGS2, and MAPK1 had statistical differences in lung squamous cell carcinoma (LUSC) compared with normal tissue (*p* < 0.001). In lung adenocarcinoma (LUAD), the expression of TP53, CASP3, MMP9, AKT1, and PTGS2 had statistical differences compared with normal tissue (*p* < 0.001), while the expression of MAPK1 had no statistical difference (*p* > 0.05). The results of survival analysis of key genes showed that AKT1, MAPK1, CASP3, MMP9, TP53, and PTGS2 had statistical differences in the OS or RFS of NSCLC patients (*p* < 0.05). In addition, the results of molecular docking indicated that the key genes and the main components have good docking activity.

**Conclusions:**

This study revealed the potential mechanism of ADI in the treatment of NSCLC with multipathways and multitargets and provided a scientific basis for the in-depth study of ADI in the treatment of NSCLC.

## 1. Introduction

Lung cancer is the leading cause of cancer-related deaths worldwide, with the 5-year survival less than 21% [1]. Nonsmall cell lung cancer (NSCLC) accounts for 85% of all lung cancers. Lung squamous cell carcinoma (LUSC) and lung adenocarcinoma (LUAD) are the two main pathological types of NSCLC [2]. In the early stages of lung cancer, it can be cured by surgery, and the treatment methods in the middle and late stages mainly include chemotherapy, radiotherapy, targeted therapy, and immunotherapy. However, since NSCLC generally has no obvious clinical symptoms in the early stage, it is often discovered during the chest impact examination, leading most patients to seek medical attention. It was already in the middle and late stages, and the opportunity for surgery was lost. With the successive identification of oncogenic driver genes in lung cancer series, molecular targeted therapy plays an important role in the treatment of advanced NSCLC, which has greatly improved the prognosis of people with positive driver genes. In recent years, with the major breakthrough of immunotherapy in the field of lung cancer, the 5-year overall survival of some patients with negative driver genes has been significantly extended. However, due to the obvious adverse reactions of molecular targeted therapy and immunotherapy [3], easy drug resistance and high medical costs made it is still a refractory disease worldwide. At present, there are still many deficiencies in the conventional treatment of NSCLC. Therefore, it is urgent to study the molecular mechanisms of NSCLC and find efficient and safe therapeutic drugs. In recent years, there have been more and more clinical trials and basic researches on the treatment of NSCLC with traditional Chinese medicine (TCM), which providing new ideas and methods for NSCLC treatment.

Aidi injection (ADI) is a compound preparation injection of Chinese herbs (Z52020236, CFDA), which is extracted from Mylabris (Banmao), Panax Ginseng (Renshen), Acanthopanax Senticosus (Ciwujia), and Astragali Radix (Huangqi), four traditional Chinese herbs [4]. In recent years, ADI combined with other antitumor drugs has been widely used in the treatment of NSCLC and has achieved good clinical effects. For example, ADI combined with chemotherapy (pemetrexed disodium and cisplatin or gemcitabine and cisplatin) had a positive effect in the treatment of NSCLC, which could effectively improve the Karnofsky performance status of patients, reduce the risk of adverse reactions, and improve the survival rate [5, 6]. In addition, studies showed that ADI combined with gefitinib had significant efficacy and high safety in the treatment of NSCLC [7, 8]. Although the clinical efficacy of ADI is remarkable, its mechanism is still unclear and needs to be further explored.

Emphasizing the holistic concept when treating diseases with TCM, as a TCM injection preparation, the function of ADI naturally depends on the mutual coordination and complementation of each Chinese herb. In order to comprehensive inquiry the effect of ADI and reveal the mechanism of ADI in the treatment of NSCLC, we employed a network pharmacology strategy. Network pharmacology is a novel discipline based on the theory of systems biology, analyzing the network of biological systems and selecting specific signaling nodes for multitargets drug molecular design [9]. Its research concept coincides with the holism of TCM and is widely used for the discovery of drugs and active compounds in TCM and the interpretation of the all-sided mechanism, providing new scientific and technological support for clinical rational drug use and advancing drug research.

## 2. Materials and Methods

### 2.1. Potential Targets of ADI

The active ingredients and related targets of Panax Ginseng, Astragali Radix, and Mylabris were obtained from the TCMSP (https://tcmspw.com/tcmsp.php) database. The active ingredients of senticosus were obtained from the TCMID (https://www.megabionet.org/tcmid/) database and then imported back into the TCMSP database one by one to obtain the related targets of senticosus. Screening conditions are oral bioavailability (OB) ≥30% and drug likeness (DL) ≥0.18.

### 2.2. NSCLC Related Targets

The NSCLC-related targets were obtained from the OMIM (https://omim.org/) and GeneCards Suite (https://www.genecards.org) databases, using nonsmall cell lung cancer or NSCLC as the search term. Merge all targets from the two databases and delete duplicate targets as the final target for NSCLC.

### 2.3. Intersecting Targets of ADI and NSCLC

In order to obtain the intersecting targets and make a Venn diagram of ADI and NSCLC, the related targets of ADI and NSCLC obtained above were inputted into the Venny 2.1.0 (https://bioinfogp.cnb.csic.es/tools/venny/index.html).

### 2.4. Construction of DDCT and PPI Network Diagrams

Running the prepared Cyto.pl script through the Perl program to obtain the linkage network file and classification file of nodes, the active ingredient file and the file of the intersecting targets of ADI and NSCLC, and he above files were imported into Cytoscape software (version 3.7.2) to build the DDCT network diagram. The PPI linkage file was obtained by using the intersecting targets of ADI and NSCLC in the STRING database (https://string-db.org). We imported the PPI linkage file into Cytoscape software to create a PPI network diagram and screened out targets with a degree and betweenness centrality (BC) greater than their mean values as the key targets for the study.

### 2.5. GO and KEGG Enrichment Analysis

The selected key targets were entered into the DAVID database (https://david.ncifcrf.gov/) for GO and KEGG enrichment analysis, and the species selected was “Homo sapiens.” The cluster profiler package and the ggplot2 data package [10] were used to perform statistical analysis and visualization for the key targets through R software (Version 3.6.3), and the main biological processes and signaling pathways involved in the treatment of NSCLC by ADI were screened out with *p* adjust <0.05. Genes in major signaling pathways were regarded as key genes for further study.

### 2.6. Expression Analysis of Key Genes

In order to explore the expression differences of the screened key genes in tumor tissue and normal tissue, we downloaded the Level 3 RNAseq data in HTSeq-FPKM format of key genes in LUSC and LUAD from the TCGA (https://portal.gdc.cancer.gov/) database. Then, the RNAseq data were converted in FPKM format to TPM format and log2 conversion. The R (3.6.3) software and ggplot2 package were used for statistical analysis and visualization [11].

### 2.7. Survival Analysis of Key Genes

To validate the results of network pharmacology, we used a survival analysis method to investigate whether the expression of key genes had an effect on survival rate with OS (overall survival) and RFS (relapse-free survival) as study indicators. The Kaplan–Meier plotter (https://kmplot.com/analysis/) was used for this study, which is a powerful online tool for assessing the correlation between the expression and survival of 30,000 genes in more than 25,000 samples from 21 tumor types [12]. We split patients by median and automatically selected the best cutoff. Survival analysis curves were plotted by a Kaplan–Meier plotter, and the log-rank*p* < 0.05 was considered statistically significant.

### 2.8. Molecular Docking

To further verify the above results, we performed molecular docking on key genes. To further verify the above results, we selected the top 10 active compounds in the DDCT network and key genes in the main signaling pathway for molecular docking. The structures of the related active compounds and key genes were obtained from PubChem (https://pubchem.ncbi.nlm.nih.gov) and the RSCB PDB database (https://www.rcsb.org/), respectively. AutoDock Vina software (https://vina.scripps.edu/) was used to perform molecular docking of key proteins and active ingredients, and the affinity value represents the binding energy of both. It is generally believed that the lower the binding energy, the more stable the binding of active ingredients and proteins [13]. The best conformation of affinity was selected as the final docking conformation and finally plotted the best results obtained using PyMol software.

### 2.9. Statistical Methods

The cluster profiler and ggplot2 data packages of R software (Version 3.6.3) were used to perform statistical analysis on the results of enrichment analysis and gene expression differences. To compare survival curves, we used the log-rank test to calculate the HR and log-rank*p* value in the Kaplan-Meier plotter. All results were considered statistically significant when *p* < 0.05, if not especially noted.

## 3. Results

**Figure 1:**
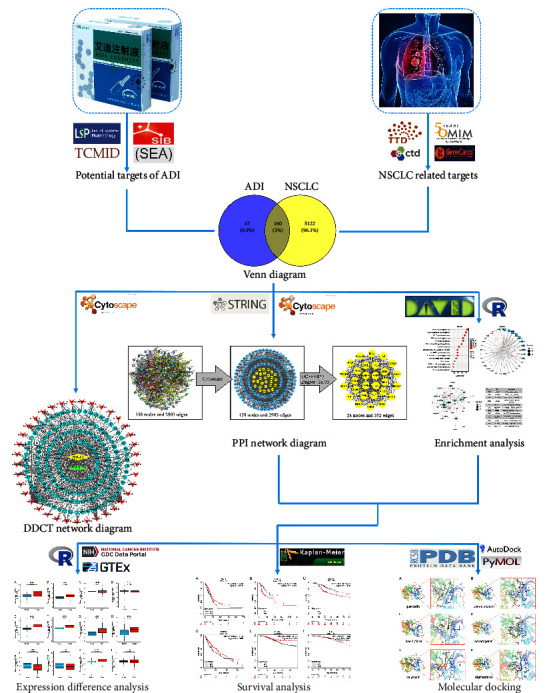
The workflow chart of this study. ADI, Aidi injection; NSCLC, nonsmall cell lung cancer; PPI, protein-protein interaction; DDCT, disease-drugcomponents-targets.

In this study, network pharmacology was used to analyze the potential targets of ADI and NSCLC, and the Venn diagram was used to obtain intersecting targets for ADI and NSCLC at the same time. Next, to identify key targets, the intersecting targets of ADI and NSCLC were used to construct a network diagram of disease-drugcomponents-targets (DDCT) and protein-protein interactions (PPI). Then, we obtained the main biological functions and signaling pathways involved in the ADI treatment of NSCLC through key targets. In order to verify the targets and signaling pathways obtained in previous, we performed differential expression analysis and survival analysis of key genes in mainly signaling pathways. Finally, the molecular docking technique was used to further verify the above results. The workflow chart of this study is shown in Figure 1.

### 3.1. Active Compounds and Targets of ADI

Through screening, a total of 37 active compounds (Table 1) and 207 targets of ADI (Supplementary Material 1) were obtained finally. Among the 37 active compounds, 15 came from Astragali Radix, 16 came from Panax Ginseng, 5 came from Acanthopanax Senticosus, and 1 came from Mylabris. The main active compounds were quercetin, formononetin, kaempferol, beta-sitosterol, stigmasterol, calycosin, isorhamnetin, methylnissolin, hederagenin, and 7-O-methylisomucronulatol. In these active compounds, some of them come from a variety of TCM.

### 3.2. NSCLC Related Targets

By searching each database, combining the results of each database and removing duplicates, a total of 5282 targets of NSCLC (Supplementary Material 2) were obtained finally.

### 3.3. Intersecting Targets of ADI and NSCLC

**Figure 2:**
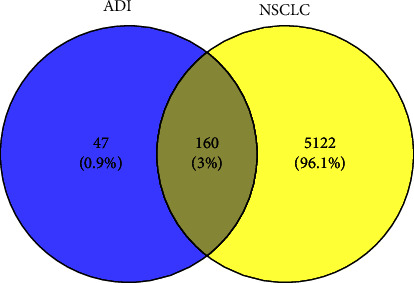
Venn diagram of components and disease related targets.

Two hundred and seven drug targets and 5,282 disease targets were imported into Venny 2.1.0, and a total of 160 intersecting targets of ADI and NSCLC (Supplementary Material 3) were obtained finally. The Venn diagram of ADI and NSCLC is shown in Figure 2.

### 3.4. DDCT and PPI Network Diagrams

**Figure 3:**
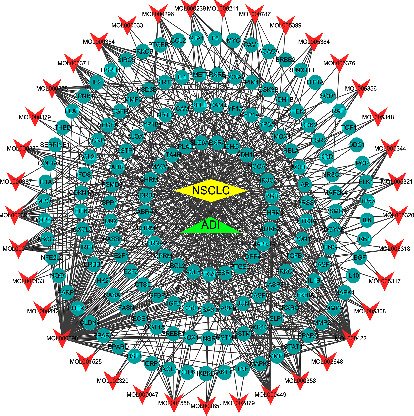
DDCT network diagram. The arrows represent active compounds; the circles represent potential targets; the diamonds represent disease; the triangles represent drug. DDCT, disease-drug-components-targets.

**Figure 4:**
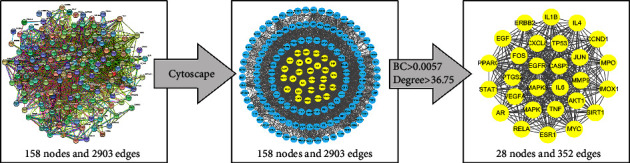
PPI network diagram. PPI, protein-protein interaction; BC, betweenness centrality.

There are a total of 199 nodes and 650 interacting lines in the DDCT network diagram (Figure 3, Supplementary Material 4). Among the 199 nodes, 37 were active ingredients, 160 were targeted proteins, one was a disease name node, and one was a drug name node. The top 10 compounds of degrees in the DDCT network diagram are shown in Table 2. The PPI network graph was obtained from the STRING database, which contained 158 nodes and 2903 interacting lines. We imported the PPI network graph file into Cytoscape software, screened out the nodes whose degree and BC were greater than their average, and finally obtained 28 key targets and 352 connecting lines (Table 3). The PPI diagram of key targets is shown in Figure 4.

### 3.5. GO and KEGG Enrichment Analysis

**Figure 5:**
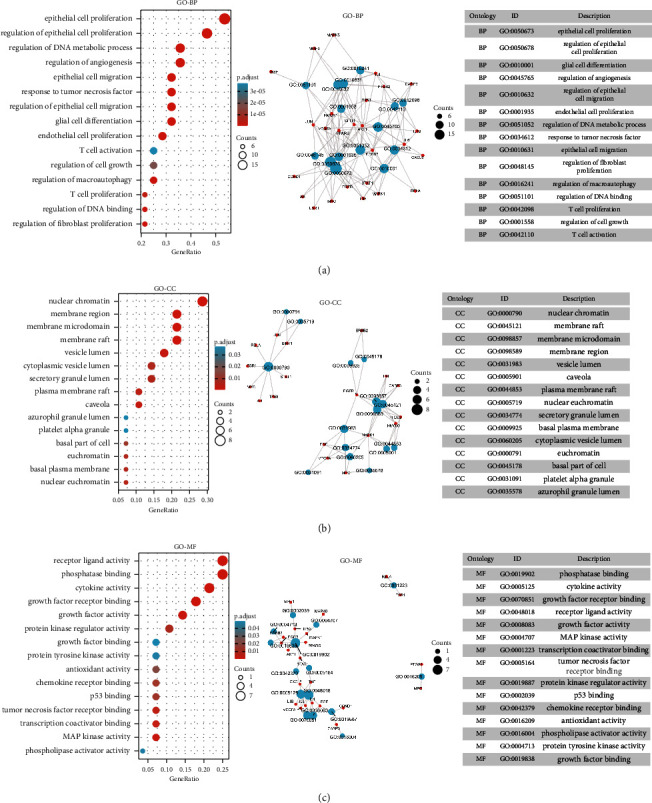
The results of GO enrichment analysis. GO, gene ontology; BP, biological process; CC, cellular component; MF, molecular function.

**Figure 6:**
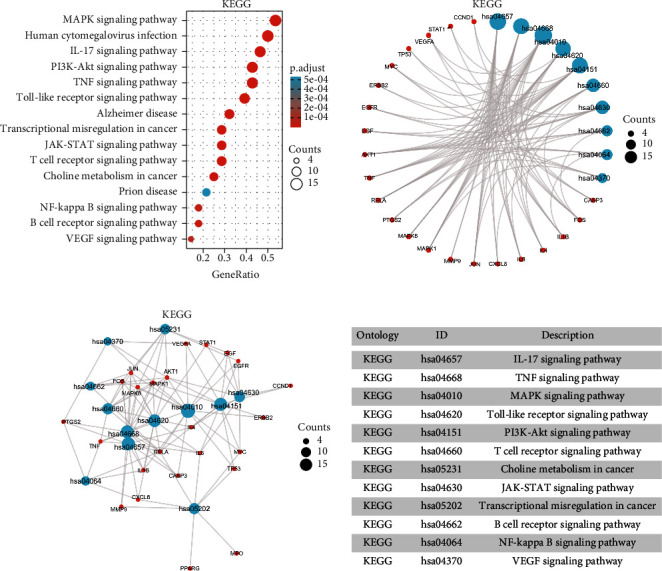
The results of KEGG enrichment analysis. KEGG, Kyoto Encyclopedia of Genes and Genomes.

GO enrichment analysis mainly describes genes from three aspects, namely, biological process (BP), cellular component (CC), and molecular function (MF). A total of 1850 entries with *p* < 0.05 of GO enrichment analysis were obtained finally, which included 1752 BP entries, 21 CC entries, and 77 MF entries (Supplementary Material 5). The 15 entries closely related to the disease were selected for visualization, respectively. As is shown in Figure 5(a), the results of BP mainly involved epithelial cell proliferation and regulation, regulation of angiogenesis, regulation of the DNA metabolic process, and regulation of macro-autophagy. CC mainly included nuclear chromatin, membrane microdomain, membrane region, and vesicle lumen (Figure 5(b)). MF mainly contained phosphatase binding, cytokine activity, growth factor receptor binding, and growth factor activity (Figure 5(c)). A total of 145 entries with *p* < 0.05 of KEGG enrichment analysis were screened (Supplementary Material 6). The results of KEGG enrichment analysis indicated that multiple signaling pathways were associated with NSCLC, which included the MAPK signaling pathway, the IL-17 signaling pathway, and PI3K/AKT signaling pathway, etc. The difference in the MAPK signaling pathway is the most obvious. The 15 entries that were closely related to NSCLC were selected for visualization (Figure 6). The key genes in these signaling pathways mainly included TP53, AKT1, CASP3, MMP9, MAPK1, and PTGS2. The results of the above enrichment analysis suggested that ADI plays an anti-NSCLC role by affecting cell apoptosis, proliferation, migration, and up-regulating or down-regulating the expression of key genes in these signaling pathways.

### 3.6. Differently Expressed Analysis of Key Genes

**Figure 7:**
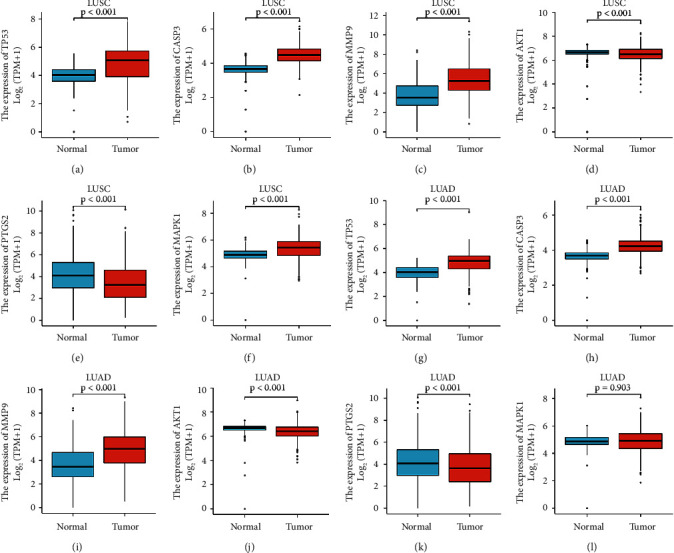
The results of key gene expression analysis. LUSC, lung squamous cell carcinoma; LUAD, lung adenocarcinoma.

The results of the differently expressed analysis of key genes showed that TP53, CASP3, MMP9, AKT1, PTGS2, and MAPK1 had statistical differences in LUSC compared with normal tissue (*p* < 0.001) (Figures 7(a)–7(f)). In LUAD, the expression of TP53, CASP3, MMP9, AKT1, and PTGS2 had statistical differences compared with normal tissue (*p* < 0.001) (Figures 7(g)–7(k)), while, the expression of MAPK1 had no statistical difference (*p* > 0.05) (Figure 7(l)). Compared with normal tissues, TP53, CASP3, and MMP9 were up-regulated in LUSC and LUAD (Figures 7(a)–7(c) and 7(g)–7(i)). Similarly, MAPK1 was up-regulated in LUSC compared with normal tissues (Figure 7(f)). Conversely, AKT1 and PTGS2 were down-regulated in LUSC and LUAD compared with normal tissues (Figures 7(d)–7(e) and 7(j)–7(k)). These results indicated that ADI exerted its therapeutic effect by affecting the expression of these key genes in tumor tissue.

### 3.7. Survival Analysis of Key Genes

**Figure 8:**
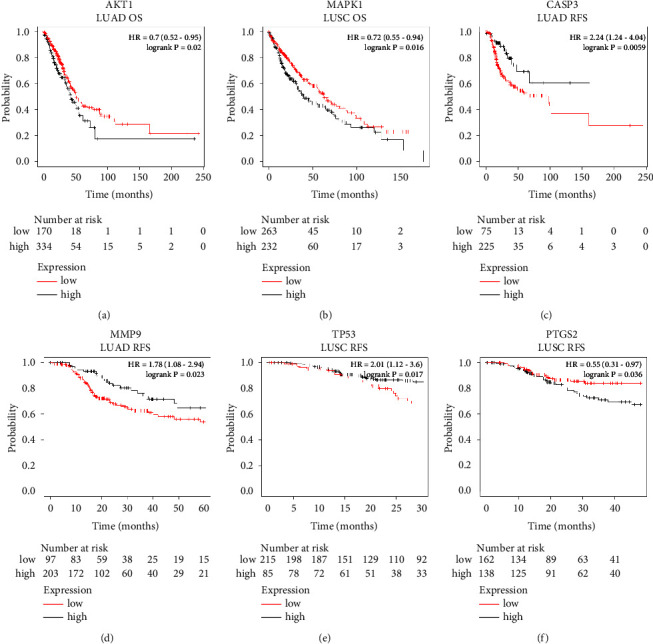
The results of key genes survival analysis. LUSC, lung squamous cell carcinoma; LUAD, lung adenocarcinoma; OS, overall survival; RFS, relapse-free survival.

In our study, the results of the survival analysis of key genes showed that AKT1, MAPK1, CASP3, MMP9, TP53, and PTGS2 had statistical differences in the OS or RFS of NSCLC patients (*p* < 0.05) (Figure 8). Patients with higher expression of AKT1 had longer OS in LUAD (Figure 8(a)), and MAPK1 had longer OS in LUSC (Figure 8(b)). Patients with lower expression of CASP3 and MMP9 had longer RFS in LUAD (Figures 8(c) and 8(d)), and similarly, in LUSC, patients with lower expression of TP53 had longer RFS (Figure 8(e)). Conversely, patients with higher expression of PTGS2 had longer RFS in LUSC (Figure 8(f)). The results of the above survival analysis were consistent with the results of the differential analysis of key gene expression, which further illustrated the accuracy of the results of this study.

### 3.8. Molecular Docking

**Figure 9:**
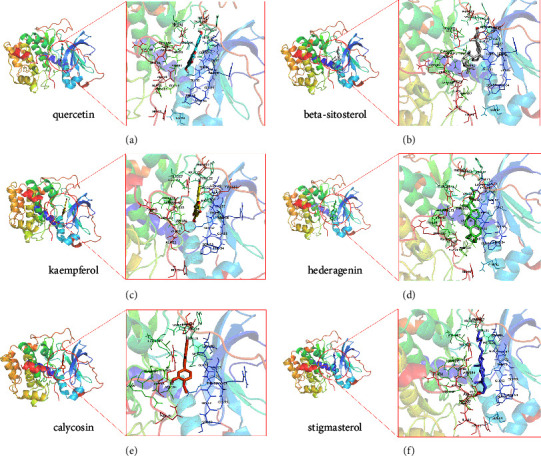
Molecular docking pattern diagram of the top 6 binding energies. (a) Quercetin and AKT1, (b) beta-sitosterol and AKT1, (c) kaempferol and AKT1, (d) hederagenin and AKT1, (e) calycosin and AKT1, and (f) stigmasterol and AKT1.

To further verify the accuracy of the results, we performed molecular docking technique on the top 10 compounds in terms of degree in the DDCT network diagram and the top 5 key genes (AKT1, CASP3, MAPK1, PTGS2, and TP53). As shown in Table 4, the results of molecular docking indicated that the lowest binding energy was stigmasterol and AKT1 with a binding energy was −10 kcal/mol. The most stable binding to CASP3 was kaempferol, and the binding energy was −8.3 kcal/mol. MAPK1 and stigmasterol had the lowest binding energy, which was stigmasterol. The most stable compounds that bind to PTGS2 and TP53 were quercetin and isorhamnetin, with binding energies of −7.5 kcal/mol and −5.8 kcal/mol, respectively. In addition, we also found that the top 6 binding energies from low to high were AKT1 and stigmasterol, quercetin, kaempferol, hederagenin, calycosin, and beta-sitosterol, respectively, which demonstrated that AKT1 may be a very critical target for ADI in the treatment of NSCLC. These results showed that the core compounds and key genes had good binding activity, which further verified that ADI exerted its therapeutic effect through these key genes. The top 6 molecular docking patterns in terms of binding energies were shown in Figure 9.

## 4. Discussion

NSCLC has no obvious clinical symptoms, and most NSCLC patients are in the advanced stage once diagnosed, with poor prognosis, which seriously threatens human health. In recent years, there have been more and more clinical trials and basic researches on ADI in the treatment of NSCLC, providing a new method for the treatment of NSCLC. Several studies have shown that ADI combined with conventional western medicine treatment can significantly improve clinical efficacy and reduce the occurrence of adverse reactions [14–16]. In order to promote the visualization and evidence-based research and development of the mechanism, this study systematically analyzed the potential mechanism of ADI in the treatment of NSCLC by using bioinformatics methods.

We found that the main components of ADI were quercetin, formononetin, kaempferol, beta-sitosterol, and stigmasterol by using the method of network pharmacology. Basic research showed that quercetin could trigger BCL2/BAX-mediated apoptosis, necrosis, and mitotic mutation, and inhibit the migration potential of A549 cells, showing good anti-NSCLC tumor activity [17]. Formononetin inhibited tumor growth by inhibiting the EGFR-Akt-Mcl-1 axis in NSCLC [18]. In addition, kaempferol could induce apoptosis of NSCLC cells by down-regulating the expression of Nrf2 [19]. Gourav Kumar found that beta-sitosterol has good anticancer activity [20]. Studies illustrated that stigmasterol effectively inhibited the activity of A549 nonsmall cell lung cancer [21]. All the above studies had demonstrated that the main components of ADI could exert its antitumor effect via inhibiting the growth of NSCLC tumors or promoting tumor cell apoptosis.

Through the PPI network map, we found that there were 28 key targets of ADI in the treatment of NSCLC, including 6 key genes that existed in the main signaling pathways, which were AKT1, TP53, CASP3, MAPK1, MMP9, and PTGS2. AKT1 was a member of the AKT kinase family, and research showed that AKT1 plays an antimetastatic role in the NSCLC cells with KRAS or EGFR mutations [22]. In addition, AKT1 down-regulation could also increase the sensitivity of NSCLC cells to chemotherapeutic agents [23]. The TP53 mutation is one of the most common mutated genes in human lung cancer. A large number of studies have shown that TP53 mutation was not only a poor prognostic factor for NSCLC but also increases the resistance of patients with EGFR mutation to EGFR-TKI therapy [24–26]. CASP3, which played a crucial role in apoptosis, had been well known. However, it had been found that CASP3 was also an oncogene in recent studies. Hence, target therapy of CASP3 not only increased the sensitivity of cancer cells to chemotherapy and radiotherapy but also inhibited the invasion and metastasis of cancer cells [27, 28]. MAPK1 could mediate cell proliferation and metastasis [29, 30]. The MAPK1/ERK2 pathway was activated in the early stage of lung cancer formation [31], which suggested that MAPK1 is a potentially important target for the early prevention and treatment of NSCLC. In addition, overexpression of MMP9 led to the occurrence of NSCLC, and inhibition of MMP9 could prevent the growth, migration, and invasion of NSCLC cells, so MMP9 is expected to become a therapeutic target for the prevention of NSCLC metastasis [32–34]. Studies have also shown that the mechanism of chemotherapy resistance in NSCLC was related to PTGS2 [35]. The results of these studies confirmed that these key targets were closely related to nonsmall cell lung cancer, which was consistent with the results of the differential expression analysis of key genes in this study.

The results of KEGG enrichment analysis indicated that multiple signaling pathways were involved in the ADI treatment of NSCLC, which included the MAPK signaling pathway, the IL-17 signaling pathway, and PI3K/AKT signaling pathway, etc. Mitogen-activated protein kinase (MAPK) is an important transmitter of signals from the cell surface to the nucleus, which is a group that can be activated by different extracellular stimuli, such as cytokines, neurotransmitters, hormones, cell stress, and cell adhesion. The basic component of the MAPK pathway is a tertiary kinase pattern that is conserved from yeast to humans, including MAP kinase kinase kinase (MKKK), MAP kinase kinase (MKK), and MAPK, which can be activated in turn to jointly regulate cells growth, differentiation and response to inflammation and other important cellular physiological or pathological processes [36]. MAPK signaling pathway is one of the common junction pathways of cell proliferation, stress, inflammation, differentiation, functional synchronization, transformation, apoptosis, and other signal transduction pathways. It transmits extracellular signals to cells through receptors, G proteins, protein kinases, and transcription factors, and participates in cell proliferation, differentiation, canceration, metastasis and apoptosis, etc. [37]. Besides, several studies have shown that the MAPK signaling pathway played a vital role in multicell processes; it is closely related to tumors [38, 39]. Overexpression of an epithelial membrane protein could inhibit the growth, migration, and invasion of NSCLC tumor cells, while knockdown of an epithelial membrane protein can enhance cell migration. Besides, epithelial membrane protein overexpression was closely related to the MAPK signaling pathway [40]. It was well known that the overexpression of ETS-homologous factor was associated with poor prognosis of NSCLC patients, and studies shew that the overexpression of ETS-homologous factor caused the occurrence of NSCLC through the AKT signaling pathway and the MAPK/ERK signaling pathway [41]. The TNF signaling pathway could not only induce the occurrence of NSCLC but also promote tumor cell proliferation and metastasis [42]. It was confirmed that CD47 upregulation in refractory lung tumor models was mediated by the TNF-*α*/NF-*κ*B1 signaling pathway, and blocking CD47 could enhance anti-tumor effects [43]. Several studies had shown that the PI3K/AKT signaling pathway, not only promoted the proliferation and migration of NSCLC cells but also increased the resistance of NSCLC to cisplatin [44]. Therefore, the PI3K/AKT signaling pathway was also a very important for the treatment of NSCLC. MiR-496 suppresses tumorigenesis via targeting the BDNF-mediated PI3K/Akt signaling pathway in NSCLC had been confirmed [45]. Dieckol inhibiting NSCLC cell proliferation and migration by regulating the PI3K/AKT signaling pathway had also been found [46]. It was well known that immune cells were closely related to the occurrence and development of NSCLC [47]. T cells and B cells are important immune cells, and studies showed immunotherapy targeting T cells or B cells was a very important method of treatment for NSCLC [48, 49]. Therefore, ADI may play a therapeutic role through the T cell receptor signaling pathway or the B cell receptor signaling pathway. NF-*κ*B was widely used by eukaryotic cells as a gene regulator to control cell proliferation and cell survival, which can regulate anti-apoptotic genes and play an indispensable role in the process of apoptosis. Research showed that miR-449a suppresses tumor growth, migration, and invasion in NSCLC by targeting the HMGB1-mediated NF-*κ*B signaling pathway [50]. VEGF was a highly specific vascular endothelial cell growth factor that promoted vascular permeability, vascular endothelial cell migration, proliferation, and angiogenesis, providing nutrients and excreting metabolites for tumor growth. Therefore, inhibiting the activation of the VEGF signaling pathway was a very important approach in the treatment of NSCLC. Bevacizumab, the world's first VEGF inhibitor, had been widely used in the treatment of NSCLC [51, 52]. The signaling pathways involved in all the above research results were closely related to NSCLC, which demonstrated the accuracy of the enrichment analysis results in this study.

It is necessary to use the method of survival analysis for the analysis of the occurrence, development, and prognosis of chronic diseases, especially malignant tumors. OS and RFS are two important reference indicators to describe the survival rate. In our study, the results of survival analysis showed that the expression of key genes in the signaling pathway had a significant impact on the OS or RFS of NSCLC patients, which verified the accuracy of the network pharmacology results, and indicated that the antitumor effect of Aidi injection was by upregulating or downregulating the expression of these key genes in tumor tissue. Molecular docking results showed that the main components of ADI and the key genes in the signaling pathway had good binding activity. It is well known that AKT1 plays an indispensable role in regulating tumor cell proliferation and apoptosis [53]. Interestingly, from the results of molecular docking, we found that the genes involved in the top 6 binding energies are all AKT1. Besides, the components with the lowest binding energies to CASP3, MAPK1, PTGS2, and TP53 were kaempferol, stigmasterol, quercetin, and isorhamnetin. CASP3 as an apoptosis-related gene played a central role in executing cell apoptosis and thus in carcinogenesis [54]. Both MAPK1 and PTGS2 were involved in cell proliferation and invasion, which made them closely related to tumorigenesis [55]. TP53 is well-known as a tumor suppressor gene that induces cell cycle arrest, apoptosis, or senescence [56]. These results demonstrated that the main components of ADI work together to exert antitumor effects by inhibiting tumor cell growth, invasion, and metastasis and promoting tumor cell senescence and apoptosis, which perfectly interpreted the overall concept of TCM.

## 5. Conclusion

Our results revealed the mechanism of ADI in the treatment of NSCLC may be through the targets of AKT1, TP53, CASP3, MAPK1, and PTGS2, regulating the MAPK signaling pathway, PI3K-AKT signaling pathway, and TNF signaling pathway to exert its therapeutic effect. In conclusion, this study revealed the mechanism of ADI against NSCLC through multipathways and multitargets, which provided new insights for clarifying the molecular mechanism of ADI and also provided a reference for the research and development of anti-NSCLC drugs.

## Figures and Tables

**Table 1:** Basic information of active ingredients of ADI.

Molecule ID	Molecule name	OB (%)	DL	Drug
MOL000098	Quercetin	46.43	0.28	Astragali Radix, Senticosus
MOL000422	Kaempferol	41.88	0.24	Panax Ginseng, Astragali Radix
MOL000392	Formononetin	69.67	0.21	Astragali Radix
MOL000378	7-O-Methylisomucronulatol	74.69	0.3	Astragali Radix
MOL000379	9,10-Dimethoxypterocarpan-3-O-*β*-D-glucoside	36.74	0.92	Astragali Radix
MOL000387	Bifendate	31.1	0.67	Astragali Radix
MOL001525	Daucosterol	36.91	0.75	Senticosus
MOL000433	FA	68.96	0.71	Astragali Radix
MOL000439	Isomucronulatol-7,2′-di-O-glucoside	49.28	0.62	Astragali Radix
MOL000442	1,7-Dihydroxy-3,9-dimethoxy pterocarpene	39.05	0.48	Astragali Radix
MOL000449	Stigmasterol	43.83	0.76	Panax Ginseng
MOL001851	3-Phenyl-4-azafluorene	32.9	0.23	Mylabris
MOL000417	Calycosin	47.75	0.24	Astragali Radix
MOL009047	Pinoresinol dimethyl ether	33.29	0.62	Senticosus
MOL000354	Isorhamnetin	49.6	0.31	Astragali Radix
MOL000371	Methylnissolin	53.74	0.48	Astragali Radix
MOL000296	Hederagenin	36.91	0.75	Astragali Radix
MOL000358	*β*-Sitosterol	36.91	0.75	Panax Ginseng
MOL005308	Aposiopolamine	66.65	0.22	Panax Ginseng
MOL005317	Deoxyharringtonine	39.27	0.81	Panax Ginseng
MOL005318	Dianthramine	40.45	0.2	Panax Ginseng
MOL005320	Arachidonate	45.57	0.2	Panax Ginseng
MOL005344	Ginsenoside Rh2	36.32	0.56	Panax Ginseng
MOL005348	Ginsenoside Rh4_qt	31.11	0.78	Panax Ginseng
MOL005356	Girinimbin	61.22	0.31	Panax Ginseng
MOL005384	Suchilactone	57.52	0.56	Panax Ginseng
MOL005399	Alexandrin_qt	36.91	0.75	Panax Ginseng
MOL001558	Sesamin	56.55	0.83	Senticosus
MOL000239	Jaranol	50.83	0.29	Astragali Radix
MOL000787	Fumarine	59.26	0.83	Panax Ginseng
MOL003648	Inermin	65.83	0.54	Panax Ginseng
MOL005321	Frutinone A	65.9	0.34	Panax Ginseng
MOL005376	Panaxadiol	33.09	0.79	Panax ginseng
MOL002320	*γ*-Sitosterol	36.91	0.75	Senticosus
MOL002879	Diop	43.59	0.39	Panax Ginseng
MOL000211	Mairin	55.38	0.78	Astragali Radix
MOL000380	(6aR, 11aR)-9,10-Dimethoxy-6a,11a-dihydro-6H-benzofurano[3,2-c] chromen-3-ol	64.26	0.42	Astragali Radix

*Note.* ADI, Aidi injection; OB, oral bioavailability; DL, drug likeness.

**Table 2:** Basic information of the top 10 compounds of degree in the DDCT network diagram.

Molecule ID	Compound	OB (%)	DL	BC	Degree
MOL000098	Quercetin	46.43	0.28	0.23996567	115
MOL000422	Kaempferol	41.88	0.24	0.03285395	81
MOL000392	Formononetin	69.67	0.21	0.01052332	24
MOL000378	7-O-Methylisomucronulatol	74.69	0.3	0.00867749	23
MOL000358	Beta-sitosterol	36.91	0.75	0.0102198	21
MOL000449	Stigmasterol	43.83	0.76	0.00886907	19
MOL000417	Calycosin	47.75	0.24	0.00398618	17
MOL000354	Isorhamnetin	49.6	0.31	0.00428171	17
MOL000371	Methylnissolin	53.74	0.48	0.00302409	14
MOL000296	Hederagenin	36.91	0.75	0.00386839	13

*Note.* DDCT, disease-drug-components-targets; OB, oral bioavailability; DL, drug likeness; BC, betweenness centrality.

**Table 3:** Basic information of 28 key targets in the PPI network diagram.

Symbol	Target	BC	Degree
AKT1	RAC-alpha serine/threonine-protein kinase	0.00392516	27
TP53	Cellular tumor antigen p53	0.00392516	27
CASP3	Caspase-3	0.00392516	27
MAPK1	Mitogen-activated protein kinase 14	0.00392516	27
PTGS2	Prostaglandin G/H synthase 2	0.00392516	27
MMP9	Matrix metalloproteinase-9	0.00392516	27
FOS	Proto-oncogenec-Fos	0.00336175	26
JUN	Transcription factor AP-1	0.00336175	26
VEGFA	Vascular endothelial growth factor A	0.00336175	26
CXCL8	Interleukin-8	0.00336175	26
MAPK8	Mitogen-activated protein kinase 8	0.00336175	26
IL6	Interleukin-6	0.00336175	26
TNF	Tumor necrosis factor	0.00336175	26
EGF	Proepidermal growth factor	0.00336175	26
MYC	Myc proto-oncogene protein	0.00236676	26
EGFR	Epidermal growth factor receptor	0.00162794	25
CCND1	G1/S-specific cyclin-D1	0.00195049	25
PPARG	Peroxisome proliferator-activated receptor gamma	0.00237789	25
IL4	Interleukin-4	0.00237789	25
IL1B	Interleukin-1 beta	0.00194291	24
RELA	Transcription factor p65	0.00121354	24
STAT1	Signal transducer and activator of transcription 1-alpha/beta	0.00122605	24
ESR1	Estrogen receptor	0.00108404	23
SIRT1	NAD-dependent deacetylase sirtuin-1	0.00140659	23
MPO	Myeloperoxidase	0.00102897	22
ERBB2	Receptor tyrosine-protein kinase ERBB2	0.00068600	22
HMOX1	Heme oxygenase 1	0.00102897	22
AR	Androgen receptor	0.00039600	21

*Note.* PPI, protein-protein interaction; BC, betweenness centrality.

**Table 4:** Molecular docking results of the top 10 components and the top 5 genes.

Molecule ID	Compound	Binding energy (kcal/mol)				
		AKT1	CASP3	MAPK1	PTGS2	TP53
MOL000098	Quercetin	−9.7	−8.0	−7.7	−7.5	−5.6
MOL000392	Formononetin	−9.0	−7.4	−7.8	−6.6	−5.3
MOL000422	Kaempferol	−9.6	−8.3	−7.8	−7.2	−5.7
MOL000358	Beta-sitosterol	−9.1	−6.9	−8.3	−6.4	−5.0
MOL000449	Stigmasterol	−10	−7.3	−8.8	−7.1	−4.8
MOL000417	Calycosin	−9.2	−7.9	−7.7	−6.7	−5.5
MOL000354	Isorhamnetin	9.6	−8.2	−7.9	−7.4	−5.8
MOL000371	Methylnissolin	−8.7	−6.9	−7.6	−7.0	−5.0
MOL000296	Hederagenin	−9.4	−7.7	−8.0	−6.3	−5.3
MOL000378	7-O-Methylisomucronulatol	−8.2	−6.6	−7.4	−6.2	−4.6

## Data Availability

The original contributions presented in the study are included in the article/supplementary materials; further inquiries can be directed to the corresponding authors.

## Supplementary Materials

Supplementary Materials Supplementary Material 1: 207 targets of ADI. ADI, Aidi injection. Supplementary Material 2: 5282 targets of NSCLC. NSCLC, nonsmall cell lung cancer. Supplementary Material 3: 160 intersecting targets of ADI and NSCLC. ADI, Aidi injection; NSCLC, nonsmall cell lung cancer. Supplementary Material 4: basic information of 199 nodes in the DDCT network diagram. DDCT, disease-drug-components-targets. Supplementary Material 5: the results of GO enrichment analysis with *p* < 0.05. GO, gene ontology. Supplementary Material 6: the results of KEGG enrichment analysis with *p* < 0.05. KEGG, Kyoto Encyclopedia of Genes and Genomes.

## References

[1] Sung H., Ferlay J., Siegel R. L. (2021). Global Global Cancer Statistics 2020: GLOBOCAN Estimates of Incidence and Mortality Worldwide for 36 Cancers in 185 Countriesancer statistics. *CA: A Cancer Journal for Clinicians*.

[2] Rodriguez-Canales J., Parra-Cuentas E., Wistuba I. I. I. (2016). Diagnosis and Diagnosis and Molecular Classification of Lung Cancer.olecular classification of lung cancer. *Cancer Treatment and Research*.

[3] Alberg A. J., Brock M. V., Ford J. G. (2013). Epidemiology of lung cancer: diagnosis and management of lung cancer, 3rd ed: American College of Chest Physicians evidence-based clinical practice guidelines. *Chest*.

[4] Xiao Z., Wang C., Zhou M. (2019). Clinical efficacy and safety of Aidi injection plus paclitaxel-based chemotherapy for advanced non-small cell lung cancer: A meta-analysis of 31 randomized controlled trials following the PRISMA guidelines meta-analysis of 31 randomized controlled trials following the PRISMA guidelines. *Journal of Ethnopharmacology*.

[5] Wang L., Yu M., Shu C. D., Qi X. C. (2022). Efficacy of Aidi injection combined with PP regimen in the treatment of advanced non-small cell lung cancer. *Journal of clinical rational drug use*.

[6] Xu C. C. (2022). Efficacy of Aidi combined with GP regimen and sole GP regimen in the treatment of advanced non-small cell lung cancer. *China Practical Medicine*.

[7] Yuan F., Chen X., Liu L. J., Li W. (2020). Efficacy of Aidi injection combined with gefitinib tablets in the treatment of elderly non-small cell lung cancer and its effect on tumor markers. *Chinese Journal of Clinical Oncology and Rehabilitation*.

[8] Hou J. L., Zhang W. Y., Liu Y. Y. (2017). Clinical trial of gefitinib tablets combination with Aidi injection in the treatment of non-small cell lung cancer in elderly patients. *The Chinese Journal of Clinical Pharmacology*.

[9] Hu Y., Liu S., Liu W. (2022). Potential Potential Molecular Mechanism of Yishen Capsule in the Treatment of Diabetic Nephropathy Based on Network Pharmacology and Molecular Dockingolecular mechanism of yishen capsule in the treatment of diabetic nephropathy based on network pharmacology and molecular docking. *Diabetes, Metabolic Syndrome and Obesity: Targets and Therapy*.

[10] Yu G., Wang L. G., Han Y., He Q. Y. (2012). ClusterProfiler: an R package for comparing biological themes among gene clusters. *OMICS: A Journal of Integrative Biology*.

[11] Vivian J., Craft B., Goldman M. (2017). Toil enables reproducible, open source, big biomedical data analyses. *Nature Biotechnology*.

[12] Lánczky A., Győrffy B. (2021). Web-Web-Based Survival Analysis Tool Tailored for Medical Research (KMplot): Development and Implementationased survival analysis tool tailored for medical research (KMplot): development and implementation. *Journal of Medical Internet Research*.

[13] Chen G., Seukep A. J., Guo M., Seukep J., Guo M. (2020). Recent advances in molecular docking for the research and discovery of potential marine drugs. *Marine Drugs*.

[14] Wang C. Q., Zheng X. T., Chen X. F. (2021). The Optimal Adjuvant Strategy of Aidi Injection With Gemcitabine and Cisplatin in Advanced Non-small Cell Lung Cancer: A Meta-analysis of 70 Randomized Controlled Trials.ptimal adjuvant strategy of Aidi injection with gemcitabine and cisplatin in advanced non-small cell lung cancer: a meta-analysis of 70 randomized controlled trials. *Frontiers in Pharmacology*.

[15] Wang J., Li G., Yu L., Mo T., Wu Q., Zhou Z. (2018). Aidi injection plus platinum-based chemotherapy for stage IIIB/IV non-small cell lung cancer: Aidi injection plus platinum-based chemotherapy for stage IIIB/IV non-small cell lung cancer: A meta-analysis of 42 RCTs following the PRISMA guidelines meta-analysis of 42 RCTs following the PRISMA guidelines. *Journal of Ethnopharmacology*.

[16] Xiao Z., Jiang Y., Chen X. F. (2020). The Hepatorenal Toxicity and Tumor Response of Chemotherapy With or Without Aidi Injection in Advanced Lung Cancer: A Meta-Analysis of 80 Randomized Controlled Trialsepatorenal toxicity and tumor response of chemotherapy with or without Aidi injection in advanced lung cancer: a meta-analysis of 80 randomized controlled trials. *Clinical Therapeutics*.

[17] Klimaszewska-Wiśniewska A., Hałas-Wiśniewska M., Izdebska M., Gagat M., Grzanka A., Grzanka D. (2017). Antiproliferative and antimetastatic action of quercetin on A549 non-small cell lung cancer cells through its effect on the cytoskeleton. *Acta Histochemica*.

[18] Yu X., Gao F., Li W., Zhou L., Liu W., Li M. (2020). Formononetin inhibits tumor growth by suppression of EGFR-Akt-Mcl-1 axis in non-small cell lung cancer. *Journal of Experimental & Clinical Cancer Research*.

[19] Fouzder C., Mukhuty A., Kundu R. (2021). Kaempferol inhibits Nrf2 signalling pathway via downregulation of Nrf2 mRNA and induces apoptosis in NSCLC cells. *Archives of Biochemistry and Biophysics*.

[20] Kumar G., Gupta R., Sharan S., Roy P., Pandey D. M., Pandey M. (2019). Anticancer activity of plant leaves extract collected from a tribal region of India. *3 Biotech*.

[21] Mohamed G. A., Ibrahim S R., Shaala L. A. (2014). Urgineaglyceride A: a new monoacylglycerol from the Egyptian Drimia maritima bulbs new monoacylglycerol from the Egyptian Drimia maritima bulbs. *Natural Product Research*.

[22] Rao G., Pierobon M., Kim I. K. (2017). Inhibition of AKT1 signaling promotes invasion and metastasis of non-small cell lung cancer cells with K-RAS or EGFR mutations. *Scientific Reports*.

[23] Lee M. W., Kim D S., Min N. Y., Kim H. T. S., Min N. Y., Kim H. T. (2008). Akt1 inhibition by RNA interference sensitizes human non-small cell lung cancer cells to cisplatin. *International Journal of Cancer*.

[24] Jiao X. D., Qin B. D., You P., Cai J., Zang Y. S. (2018). The prognostic value of TP53 and its correlation with EGFR mutation in advanced non-small cell lung cancer, an analysis based on cBioPortal data base. *Lung Cancer*.

[25] Qin K., Hou H., Liang Y., Zhang X. (2020). Prognostic value of TP53 concurrent mutations for EGFR- TKIs and ALK-TKIs based targeted therapy in advanced non-small cell lung cancer: a meta-analysis. *BMC Cancer*.

[26] Labbé C., Cabanero M., Korpanty G. J. (2017). Prognostic and predictive effects of TP53 co-mutation in patients with EGFR-mutatednon-small cell lung cancer (NSCLC). *Lung Cancer*.

[27] Bernard A., Chevrier S., Beltjens F. (2019). Cleaved Cleaved Caspase-3 Transcriptionally Regulates Angiogenesis-Promoting Chemotherapy Resistanceaspase-3 transcriptionally regulates angiogenesis-promoting chemotherapy resistance. *Cancer Research*.

[28] Zhou M., Liu X., Li Z., Huang Q., Li F., Li C. Y. (2018). Caspase-3 regulates the migration, invasion and metastasis of colon cancer cells. *International Journal of Cancer*.

[29] Lin L., Han M. M., Wang F., Xu L. L., Yu H. X., Yang P. Y. (2014). CXCR7 stimulates MAPK signaling to regulate hepatocellular carcinoma progression. *Cell Death & Disease*.

[30] Guégan J.-P., Ezan F., Théret N., Langouët S., Baffet G. (2013). MAPK signaling in cisplatin-induced death: predominant role of ERK1 over ERK2 in human hepatocellular carcinoma cells. *Carcinogenesis*.

[31] López-Malpartida A. V., Varela G., García Pichel J. D., Varela G., García Pichel J. (2009). Differential ErbB receptor expression and intracellular signaling activity in lung adenocarcinomas and squamous cell carcinomas. *Lung Cancer*.

[32] Li W., Jia M., Wang J. (2019). Association of MMP9-1562C/T and MMP13-77Association of MMP9-1562C/T and MMP13-77A/G Polymorphisms with Non-Small Cell Lung Cancer in Southern Chinese Population/G polymorphisms with non-small cell lung cancer in southern Chinese population. *Biomolecules*.

[33] Li J., Wang H., Ke H., Ni S. (2015). MiR-129 regulates MMP9 to control metastasis of non-small cell lung cancer. *Tumor Biology*.

[34] Zhen Y., Liu J., Huang Y., Wang Y., Li W., Wu J. (2017). miR-133bmiR-133b Inhibits Cell Growth, Migration, and Invasion by Targeting MMP9 in Non-Small Cell Lung Cancernhibits cell growth, migration, and invasion by targeting MMP9 in non-small cell lung cancer. *Oncology Research Featuring Preclinical and Clinical Cancer Therapeutics*.

[35] Lin X. M., Luo W., Wang H. (2019). The The Role of Prostaglandin-EndoperoxideSynthase-2 in Chemoresistance of Non-Small Cell Lung Cancer.ole of prostaglandin-endoperoxidesynthase-2 in chemoresistance of non-small cell lung cancer. *Frontiers in Pharmacology*.

[36] Sun Y., Liu W. Z., Liu T., Feng X., Yang N., Zhou H. F. (2015). Signaling pathway of MAPK/ERK in cell proliferation, differentiation, migration, senescence and apoptosis. *Journal of Receptors and Signal Transduction*.

[37] Yue J., López J. M. (2020). Understanding MAPK Understanding MAPK Signaling Pathways in Apoptosisignaling pathways in apoptosis. *International Journal of Molecular Sciences*.

[38] Burotto M., Chiou V., Lee J. M., Kohn E. C., Lee J. M., Kohn E. C. (2014). The MAPK pathway across different malignancies: a new perspective. *Cancer*.

[39] Guo Y. J., Pan W. W., Liu S. B., Shen Z. F., Xu Y., Hu L. L. (2020). *Experimental and Therapeutic Medicine*.

[40] Ma Y., Schröder D. C., Nenkov M. (2021). Epithelial Epithelial Membrane Protein 2 Suppresses Non-Small Cell Lung Cancer Cell Growth by Inhibition of MAPK Pathwayembrane protein 2 suppresses non-small cell lung cancer cell growth by inhibition of MAPK pathway. *International Journal of Molecular Sciences*.

[41] Gao L., Yang T., Zhang S. (2021). EHF enhances malignancy by modulating AKT and MAPK/ERK signaling in non-small cell lung cancer cells. *Oncology Reports*.

[42] Ma W., Chen X., Wu X. (2020). Long noncoding RNA SPRY4-IT1 promotes proliferation and metastasis of hepatocellular carcinoma via mediating TNF signaling pathway. *Journal of Cellular Physiology*.

[43] Zhang X., Wang Y., Fan J. (2019). Blocking CD47 efficiently potentiated therapeutic effects of anti-angiogenic therapy in non-small cell lung cancer. *Journal for ImmunoTherapy of Cancer*.

[44] Yu X., Li Y., Jiang G. (2021). FGF21 promotes non-small cell lung cancer progression by SIRT1/PI3K/AKT signaling. *Life Sciences*.

[45] Ma R., Zhu P., Liu S., Gao B., Wang W. (2019). miR-496 Suppress tumorigenesis via targeting BDNF-mediated PI3K/Akt signaling pathway in non-small cell lung cancer. *Biochemical and Biophysical Research Communications*.

[46] Wang C. H., Li X. F., Jin L. F., Zhao Y., Zhu G. J., Shen W. Z. (2019). Dieckol inhibits non-small-cell lung cancer cell proliferation and migration by regulating the PI3K/AKT signaling pathway. *Journal of Biochemical and Molecular Toxicology*.

[47] Stankovic B., Bjørhovde H. A. K., Frafjord A. (2018). Immune Immune Cell Composition in Human Non-small Cell Lung Cancer.ell composition in human non-small cell lung cancer. *Frontiers in Immunology*.

[48] Reuben A., Zhang J., Chiou S. H. (2020). Comprehensive T cell repertoire characterization of non-small cell lung cancer. *Nature Communications*.

[49] Chen J., Tan Y., Sun F. (2020). Single-cell transcriptome and antigen-immunoglobin analysis reveals the diversity of B cells in non-small cell lung cancer. *Genome BiolGenome Biology*.

[50] Wu D., Liu J., Chen J., He H., Ma H., Lv X. (2019). miR-449amiR-449a Suppresses Tumor Growth, Migration, and Invasion in Non-Small Cell Lung Cancer by Targeting a HMGB1-Mediated NF-*κ*B Signaling Pathwayuppresses tumor growth, migration, and invasion in non-small cell lung cancer by targeting a HMGB1-mediated NF-*κ*B signaling pathway. *Oncology Research Featuring Preclinical and Clinical Cancer Therapeutics*.

[51] Garcia J., Hurwitz H. I., Sandler A. B. (2020). Bevacizumab (Avastin) in cancer treatment: Bevacizumab (Avastin) in cancer treatment: A review of 15 years of clinical experience and future outlook review of 15 years of clinical experience and future outlook. *Cancer Treatment Reviews*.

[52] Yang Y., Wang L., Li X. (2022). Efficacy and safety of bevacizumab combined with EGFR-TKIs in advanced non-small cell lung cancer: A meta analysis. *Thoracic Cancer*.

[53] Hinz N., Jücker M. (2019). Distinct functions of AKT isoforms in breast cancer: a comprehensive review. *Cell Communication and Signaling*.

[54] Lin J., Zhang Y., Wang H. (2016). Genetic Genetic Polymorphisms in the Apoptosis-Associated Gene CASP3 and the Risk of Lung Cancer in Chinese Populationolymorphisms in the apoptosis-associated gene CASP3 and the risk of lung cancer in Chinese population. *PLoS One*.

[55] Rizzo M. T., Rizzo T. (2011). Cyclooxygenase-2 in oncogenesis. *Clinica Chimica Acta*.

[56] D’Orazi G. (2021). Recent Recent Advances in p53dvances in p53. *Biomolecules*.

